# Reactive oxygen species and oxidative signalling in plants

**DOI:** 10.1042/EBC20250047

**Published:** 2026-07-30

**Authors:** Christine H. Foyer

**Affiliations:** School of Biosciences, College of Life and Environmental Sciences, University of Birmingham, Edgbaston B15 2TT, U.K.

**Keywords:** cell nucleus, hydrogen peroxide, redox signalling, superoxides

## Abstract

Plants mastered the art of reduction/oxidation (redox) control early in evolution, incorporating reactive oxygen species (ROS) as ubiquitous ‘pioneer’ signalling molecules that mediate growth and stress responses. Each compartment of the plant cell generates ROS, particularly superoxide and hydrogen peroxide. By virtue of their photosynthetic metabolism, plants produce large quantities of hydrogen peroxide, particularly through the process of photorespiration, but they maintain low levels of oxidant accumulation in the absence of environmental or metabolic perturbations. ROS production and signalling are intimately associated with plant biology, such as growth and development, stress resistance, and immune responses. The chemical reactivity of ROS makes them excellent local and systemic signalling molecules. ROS produced by the respiratory burst oxidase homologues are particularly important in the long-distance cell-to-cell signalling process called the ROS wave. Interactions with cell proteins, particularly ROS receptors, such as the receptor kinase called H_2_O_2_-induced Ca^2+^ increases 1, serve to modulate the network of metabolic and hormonal pathways that underpin regulation and functions. ROS-dependent post-translational modifications of protein Cys residues are a central mechanism for the transmission of redox signals. In addition, ROS promote liquid–liquid phase separation that alters the location and function of proteins such as transcription factors, facilitating the formation of heterotypic transcriptional condensates that alter gene expression. ROS-dependent phase separation is a key mechanism for plants to adapt to environmental changes.

## Introduction

Oxygen (O_2_) is an immensely versatile molecule that fulfils crucial life functions underpinning cell metabolism in most living organisms on earth. Plant cysteine oxidases (PCOs) and group VII ethylene response factors (ERFVIIs) are major components of plant hypoxia sensing through the cysteine (Cys)/Arg branch of the N-degron pathway (Cys-NDP) for protein degradation that is present in all vascular plants [1].

Part of the oxygen consumed by plants is transformed into reactive oxygen species (ROS), which include radicals such as superoxide (O_2_^•−^) and hydroxyl (OH^•^) and non-radical compounds, such as hydrogen peroxide (H_2_O_2_), each of which triggers specific redox changes and signalling pathways. Oxygen and ROS signalling are interlinked and are likely to have multiple sites of reciprocal control. For example, the reoxygenation of plant tissues after periods of hypoxia induces a ROS burst. H_2_O_2_ production facilitates stabilisation of ERFVIIs through ROS-mediated inactivation of PCOs [2].

ROS have higher chemical reactivity than ground-state molecular oxygen [3], each type of ROS molecule having different levels of relative reactivity [4]. Superoxide is formed when the dioxygen molecule (O_2_) gains a single electron in an antibonding π* orbital, creating an unpaired electron (denoted by ‘^•^’) and a net negative charge ‘^―^’. Superoxide generation is the major trigger for cellular oxidation because, once formed, the dismutation of superoxide leads to the formation of hydrogen peroxide, a reaction that is largely produced through the action of superoxide dismutases (SOD). The perception of these oxidative signals and the resultant transmission of redox changes regulate multiple plant processes, including seed germination, root meristem maintenance, root hair development, lateral root formation, shoot apical meristem activity, flowering and reproductive organ development [6–11].

Superoxide acts as both a weak base (pK_a_ ∼4.8) and a one-electron oxidant/reductant. Thus, O_2_^•−^ has a short half-life and low mobility due to its negative charge. However, its ability to reduce oxidised ferric iron (Fe^3+^) forms to ferrous iron (Fe^2+^) in the localised cellular environments within plant proteins can have significant consequences for protein functions and cell signalling. The functions of O_2_^•−^ relate to at least two factors: its ability to release Fe^2+^ from [Fe-S] proteins and ferritin and its reaction with nitric oxide radical (NO^•^) to produce peroxynitrite, ONOO^―^ (O_2_^•−^ + NO^•^ → ONOO^―^). For example, superoxide has been shown to directly regulate the glycosylase/lyase activities of the DNA demethylase repressor of silencing 1 (ROS1) in plant stem cells [5]. Superoxide-mediated activation of DNA glycosylase/lyase activity to control the stem cell niche and maintain stem cell fate [5]. The formation of hydroxyl radicals through processes such as the Fenton reaction, in which Fe^2+^ reacts with H_2_O_2_ to generate the OH^•^, is particularly important in the cell wall, where it appears to fulfil multiple functions.

Superoxide anions are removed by the action of the SOD family of enzymes, which catalyse a dismutation reaction (2 O_2_^•−^ + 2 H^+^ → O_2_ + H_2_O_2_). This reaction also occurs non-enzymatically, but at much slower rates. Superoxide has higher chemical reactivity and is more unstable than H_2_O_2_. While H_2_O_2_ has limited activity, it can oxidise certain protein cysteine residues, a process that forms the basis for ROS signalling pathways. H_2_O_2_ is the substrate for antioxidant enzymes such as catalases, ascorbate peroxidases, peroxiredoxins, and thiol peroxidases.

## Compartment-specific ROS production and signalling

**Figure 1:**
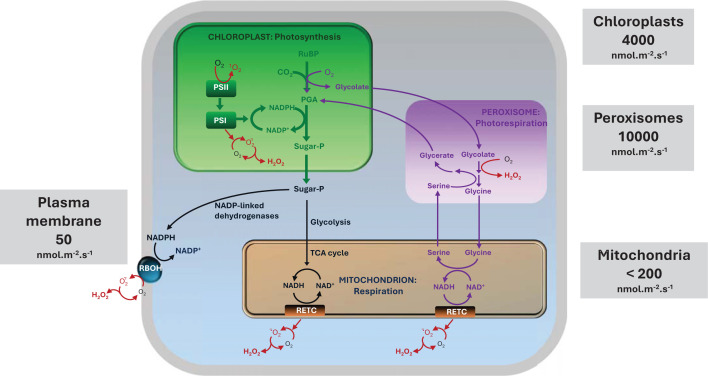
Diagrammatic representation of the main sources of reactive oxygen species (ROS) in plant cells Diagrammatic representation of the main sources of ROS in plant cells. The numbers in boxes are the calculated rates of hydrogen peroxide production in the different cellular compartments. PGA: phosphoglycerate; PSI: Photosystem I; PSII: Photosystem II; RuBP: ribulose-1,5-bisphosphate; Sugar-P: sugar phosphate; TCA: tricarboxylic acid cycle.

The photosynthetic and respiratory electron transport chains of the chloroplasts and mitochondria produce superoxide by direct electron donation to molecular oxygen. Contrary to the common misconception that plant cells produce only 1%–2% of consumed oxygen as ROS, photorespiration alone accounts for ∼70% of H_2_O_2_ production in C3 plants under standard conditions (Figure 1), with this proportion increasing to 35% of total electron flow under stress [12–14]. Other enzymes such as xanthine oxidoreductase and sulphite oxidase also generate H_2_O_2_ in the peroxisomes. Nevertheless, the steady-state levels of H_2_O_2_ accumulated in plant cells are maintained at a low level through the activity of the efficient plant antioxidant system and the thiol-based redox systems that mediate redox regulation and signalling throughout plant cells [13,14]. Precise control of cellular redox homeostasis is essential for plant responses to biotic and abiotic stresses and for the maintenance of the cellular network of metabolic and signalling processes, including phytohormone synthesis, transcriptional reprogramming and the control of cell suicide programmes.

In addition to the generation of superoxide and hydrogen, the photosynthetic electron transport system also generates singlet oxygen [15]. While singlet oxygen is produced primarily at photosystem II and light-harvesting complexes within the grana core (appressed regions) of thylakoid membranes, due to the formation of the excited triplet state of chlorophyll, which can transfer excitation energy directly to molecular oxygen during photosynthesis, it can also be generated in non-photosynthetic tissues experiencing severe stress. Singlet oxygen is an important signalling molecule that is distinct and operates independently of other ROS signalling cascades [15]. It serves important functions in the chloroplast-to-nucleus communication that regulates nuclear gene expression to trigger acclimation and/or programmed cell death (PCD). This process occurs largely through the EXECUTER1 (EX1) pathway relays, but the oxidation products of thylakoid membrane carotenoids, such as β-cyclocitral, are also recognised crucial signalling molecules that are produced because of the action of singlet oxygen [15]. While hydroxyl (OH•) radicals are the most reactive member of the ROS family due to an extremely unstable single unpaired electron, they are also considered to function in signalling; their primary role is in the regulation of cell wall loosening for growth, root elongation, and stomatal closure.

**Figure 2:**
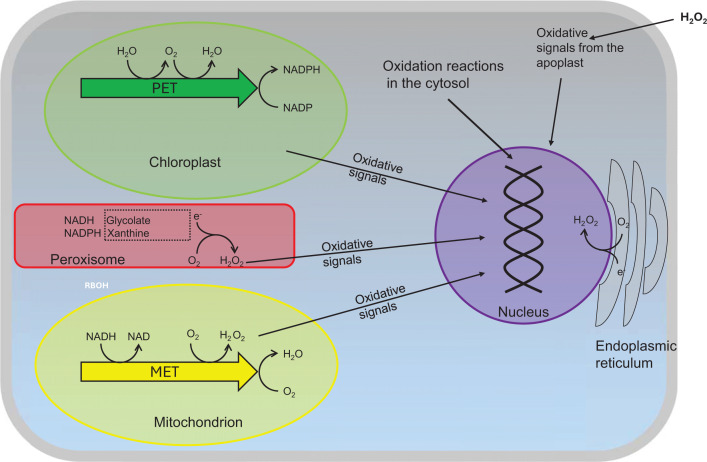
Diagrammatic representation of the transfer of oxidative signals from different sites of generation in the plant cell to the nucleus Diagrammatic representation of the transfer of oxidative signals from different sites of generation in the plant cell to the nucleus. MET: mitochondrial electron transport chain; PET: Photosynthetic electron transport chain.

Superoxide generation through electron donation to oxygen occurs in the mitochondrial electron transport chain, largely through Complex I and Complex III. Superoxide and hydrogen peroxide are generated by enzymes in all compartments of plant cells, including the cell wall/apoplast (peroxidases) and the apoplastic face of the plasma membrane via the action of respiratory burst oxidase homologues (RBOHs). Accumulating evidence suggests that superoxide is also directly generated in the nuclei of plant cells [16]. There are numerous examples of H_2_O_2_ accumulation in the nuclei of plant cells in the literature, although it is less certain whether H_2_O_2_ is transported into the nuclei from other cellular compartments or whether H_2_O_2_ is generated directly in the nuclei via the association of the nuclear envelope with RBOH proteins in the endoplasmic reticulum, or both (Figure 2). For example, H_2_O_2_ accumulation in the nuclei of roots encountering air spaces leads to the Cys- and redox-dependent multimerisation of the auxin repressor protein IAA3 and arrest of lateral root growth [17]. Such mechanisms are key components of oxidative regulation signalling, particularly in relation to plant adaptation to environmental stresses.

Since all the compartments of the plant cell produce ROS (Figure 1) and the relative rates of ROS production in each compartment can vary independently of growth [18] in response to metabolic and environmental cues in order to modulate nuclear gene expression, a key question concerns how the specificity of ROS sensing and redox signalling pathways in each compartment is achieved. For example, disruptions in photosynthesis and/or respiration in response to environmental stress increase ROS production in these compartments. Simultaneously, RBOH enzymes are activated and signal via the apoplast/plasma membrane. While it is unclear precisely how much chloroplasts and mitochondria contribute to overall ROS accumulation and inter- and intracellular cell signalling under different stress conditions, current concepts assume that each compartment makes a unique contribution to the repertoire of signals received by the nucleus. While hydrogen peroxide can move between compartments, the assumption is that each compartment has its own ROS receptors and unique signalling pathways that supply compartment-specific information to the nucleus. Moreover, each compartment has its own complement of antioxidants that police the local signal intensity. Some compartments such as the chloroplasts and peroxisomes are rich in antioxidants; others such as the endoplasmic reticulum and apoplast/cell wall have little antioxidant capacity and reside in a highly oxidised state.

## Redox-based post-translational modifications of proteins

Redox post-translational protein modifications (redox PTMs) primarily occur at Cys residues, where the sulphur atom in the sulfhydryl (thiol; −SH) group can adopt a wide range of oxidation states. Cys thiols are oxidised by H_2_O_2_ accumulation to form sulphenic acid (RSOH). This unstable intermediate can react with other reactive sulphur, oxygen or nitrogen species. These interactions lead to various PTMs, including S-sulfenylation (–SOH), disulphide bond formation (–RSSR–), S-glutathionylation (–RSSG), S-nitrosylation (–SNO), persulfidation (–RSSH), and the formation of sulfinic (–SO_2_H) or sulfonic acids (–SO_3_H) [19]. Of these, protein S-nitrosylation is the main route of nitric oxide (NO) signalling, which has been shown to modulate numerous growth and stress responses [20,21].

Cys oxidation is catalysed by enzymes, such as by protein disulphide isomerases. These enzymes play a crucial role in protein folding by facilitating the formation and rearrangement of disulphide bonds (S–S) between Cys residues. Alternatively, Cys oxidation can occur indirectly by thiol peroxidases through disulphide exchange [22]. Cys oxidations are largely reversible, through reactions that are catalysed by redoxin proteins such as thioredoxins (TRX) and glutaredoxins (GRX), which transfer reducing power from redox metabolites such as glutathione (GSH), NADPH and ascorbate [13,14].

Cys-rich, disordered regions are frequently found in proteins that sense and respond to oxidative stress, acting as thiol sensors and switches [23]. The Cys residues in the intrinsically disordered regions (IDRs) of proteins often exhibit unique functional, structural, and chemical properties that differ from those in folded, globular domains. In reducing environments, these regions are typically disordered. Under oxidising conditions, disulphide bond formation can induce structural changes that alter protein activity or binding affinity, particularly in relation to the redox-dependent formation and regulation of biomolecular condensates that act as molecular switches that control phase separation through disulphide bond formation. These residues enable proteins to form local high-concentration protein assemblies (condensates) accompanied by liquid-liquid phase separation in response to redox signals that exert strong effects on transcriptional and epigenetic regulation [24]. For example, zinc fingers 1 and 2 of lesion simulating disease 1 are part of a three-zinc-finger motif structure located at the N-terminus of the protein that regulates interactions with other proteins’ condensate formation [25]. This process plays a crucial role in the regulation of catalase distribution between the peroxisomes and nuclei and associated functions that control PCD. There is an increasing body of evidence suggesting that regulated ROS accumulation in nuclei is required for the Cys-dependent regulation of transcription factors that control plant growth, development and defence responses. For example, Cys oxidation also drives the phase separation that alters the binding of transcription factors, such as terminating flower (TMF), in tomato plants [25]. The formation of TMF biomolecular condensates allows binding to, and sequestration of, the promoter of the floral identity gene *ANANTHA* [25]. Elevated ROS levels in root nuclei trigger the redox-dependent multimerisation of the auxin repressor protein IAA3, which is required for interactions with the co-repressor TPL, thereby attenuating IAA3-mediated target gene repression that shapes root adaptive responses [17]. The accumulation of ROS in the nuclei that accompanies this regulation is dependent on the activities of RBOH enzymes [17,25].

## RBOH-mediated signalling

ROS production in the different compartments makes a major contribution to the regulation of metabolism and signalling in plant cells. The relative contribution of each compartment to ROS generation is likely to add specificity to the signal, depending on the local presence of ROS receptors and other interacting proteins, as well as antioxidants at any moment in time. The multifunctional RBOH proteins are central hubs in the ROS signalling that play crucial roles in local and systemic processes that regulate plant growth, development, and environmental stress responses [26,27]. While these proteins are localised primarily on the plasma membrane, specific RBOHs (particularly RBOHD and RBOHF) have been found to shift localisation to the endoplasmic reticulum, depending on the nature of interacting proteins [28]. For example, tobacco RBOHD was found in lipid rafts, which are membrane microdomains that can be linked to other membrane components [29]. The range of protein/protein interactions, together with PTM-dependent regulation via nitrosylation, persulfidation and acetylation, as well as recycling via clathrin-mediated endocytosis, alters RBOHs’ function [30].

In plants such as *Arabidopsis thaliana*, the RBOH protein C and other root-localised RBOHs regulate root responses to environmental triggers; RBOHD and RBOHF are essential regulators of stomatal closure and plant immune responses. These proteins also produce the cell-to-cell ROS signals that are essential components of systemic pathways leading to whole plant environmental stress responses. The RBOH enzymes physically interact with different kinases in the plasma membrane, such as the botrytis-induced kinase 1 (BIK1) that, together with different phosphatases, phosphorylate/de-phosphorylate RBOHs and regulate their enzymatic activities [28]. RBOHs interact with other proteins such as Rho-of-plants and calcium/different calcium-binding proteins [31]. Phytochrome b (phyB) also interacts with RBOHD to regulate ROS production [32]. It has been recently shown that the plasma membrane-localised receptor kinase, called Feroni, phosphorylates phyB and is involved in the regulation of ROS production in response to high-light stress [33]. This regulatory system also involves cysteine-rich receptor-like kinase 10 and the plasma membrane intrinsic protein 2;6 (PIP2;6), which interact with RBOHD and phyB to regulate RBOHD-dependent ROS production [33].

## Local and systemic signalling

A hierarchy of systemic signals works together in a complex, multi-layered, and fast-acting network to facilitate the rapid transmission of information from local stressed tissues to distal organs, enabling systemic acquired acclimation and resistance (SAR) responses [27]. Within this context, stress-induced electrical signals cause depolarisation of the plasma membrane, exciting and activating different calcium channels and causing increases in cytosolic Ca^2+^ levels that trigger RBOH-mediated ROS production [27,33–35] Mutations in the GLUTAMATE-LIKE RECEPTOR channels GLR3.3 and GLR3.6 that abolish the transfer of systemic electric signals [36,37] also block the transfer of Ca^2+/^ROS signals from cell to cell and from leaf to leaf in a self-propagating ‘ROS wave’ [26]. ROS wave signalling requires redox receptors such as the plasma membrane leucine-rich-repeat receptor kinase called H_2_O_2_-induced Ca^2+^ increases (HPCA1) [34]. HPCA1 is activated by H_2_O_2_-dependent covalent modification of extracellular Cys residues, which triggers autophosphorylation of HPCA1, in order to regulate stomatal closure and systemic stress responses [34]. This pathway also involves plasma membrane intrinsic proteins (PIPs), which are a subclass of AQPs that mediate the transport of H_2_O and other small compounds, including H_2_O_2_ [37–40]. The PIPs regulate ROS wave signalling and plant immune responses by mediating H_2_O_2_ transmembrane transport [37]. For example, AtPIP1;4 mediates the transport of flg22-induced apoplastic H_2_O_2_ into the cytoplasm, activating the MAPK cascade that promotes callose deposition and activation of the SAR pathway [38]. Similarly, AtPIP2;1 mediates H_2_O_2_ transport to regulate stomatal closure and immunity against pathogen invasion [39,40]. Protonated superoxide could potentially also transfer through PIPs or other channels in the plasma membrane, becoming deprotonated in the cytosol due to the more basic cytosolic pH.

## Conclusions and perspectives

Accumulating literature evidence supports the concept that ROS are generated by living cells, as critical ‘pro-life’ signals that function together with NO and hormone signals to modulate the network of genetic and epigenetic processes that mediate adaptation to metabolism and environmental changes [41]. However, upon the perception of extreme biotic and abiotic threats, particularly during plant immune responses, ROS mediate PCD by triggering controlled cell dismantling for development, immunity, and stress adaptation [42]. Superoxide radical anion and hydrogen peroxide are essential components of the plant redox code [43], which refers to the fundamental principles organising biological systems through redox reactions, involving electron transfers that regulate cell function, development, and adaptation. These oxidants, together with antioxidants and a plethora of redox sensor regulators, particularly TRX, PRX, and GRX [44], form a universal biological language that functions alongside the genetic code to organise cellular functions from stem cells to whole organisms in relation to environmental change. These principles can be applied to cell-to-cell and plant-to-plant signalling processes [44], in which the regulation and localisation of RBOHs play a key role. While much remains to be educated regarding the integral components of directional cell-to-cell systemic signalling and pathways, waves of ROS and Ca^2+^ signalling are interconnected in the perception and transmission of signals [44]. The putative molecular switches that connect these signalling pathways and that provide specificity to the signalling process regarding the perception of different environmental stresses to trigger appropriate responses are largely unknown.

Rather than acting randomly, hydrogen peroxide specifically modifies protein Cys residues to modify the interactome and functions of proteins. Cys oxidation can lead to the formation of redox-sensitive protein composites, such as disulphide-linked dimers or alterations in the structure and, hence, binding properties of transcription factors [17,25]. In some cases, Cys oxidations lead the assembly of higher-order protein structures such as stress granules. These dynamic, membrane-less cytoplasmic aggregates form rapidly in response to environmental stresses and are composed of messenger ribonucleoproteins, primarily untranslated mRNAs, RNA-binding proteins, and translation initiation factors [45,46].

Much remains uncertain about the redox sensors and other signalling components that transmit information from the different compartments of the cell to the nucleus and how these provide the specificity that is required to trigger transcriptional responses that are appropriate to the perceived stress. Crucially, little is known about the ability of the nucleus to generate ROS and other oxidative signals and how ROS generated by RBOH rapidly accumulate in the nucleus. Similarly, the role of protein/protein interactions and protein composites in relocating antioxidant and other enzymes to cellular compartments other than those dictated by their targeting sequences is intriguing and requires further investigation. The effects of ROS on cells are highly dependent on cell identity, as different cell types possess distinct metabolic states, antioxidant capacities, and specialised functions that determine their sensitivity or responsiveness to oxidation. While PCD is essential for plant development and defence, certain cell types exhibit high resistance to oxidation-induced cell suicide, allowing them to survive extreme stress, maintain structural integrity, or function as specialised tissues. Understanding the processors and factors that underpin such resilience will be key to improving plant performance in a changing and often unfavourable climate.

## Summary

- ROS, such as the superoxide radical anion and hydrogen peroxide, are essential integrators in plant biology. They connect the perception of environmental signals with growth, development, and defence responses through mechanisms including protein oxidation, nuclear translocation, and transcriptional regulation.
- While ROS are produced by all the compartments of plant cells, superoxide generation in the apoplast by the RBOHs is a universal instigator of oxidative/redox signalling pathways. Compartment-specific ROS receptors add specificity to the signal transduction process.
- Redox PTMs, particularly involving protein cysteine residues, which act as ROS sensors that undergo reversible oxidation to modulate protein function stability and localisation through multiple pathways.
- Redox-regulated protein/protein interactions and the formation of membrane-less compartments/composites, forming dynamic molecular environments that regulate diverse functions across the whole regulatory network.
- Future directions include identification of ROS sensors in each compartment of plant cells and the role of oxidants and antioxidants in the nucleus in regulating genetic and epigenetic responses to environmental stress.

## Open Access

Open access for this article was enabled by the participation of the University of Birmingham in an all-inclusive Read & Publish agreement with Portland Press and the Biochemical Society under an agreement with JISC.

## Abbreviations

ERFVIIs: group VII ethylene response factors
HPCA1: H_2_O_2_-induced Ca^2+^ increases 1
GRX: glutaredoxins
PIPs: plasma membrane intrinsic proteins
PIP2;6: plasma membrane intrinsic protein 2;6
PCOs: plant cysteine oxidases
PTMs: post-translational modifications
ROS: reactive oxygen species
SAR: systemic acquired resistance
TMF: terminating flower
